# Obituary—Mrs. Wm. A. Hawley

**Published:** 1889-07

**Authors:** 


### OBITUARY.

### MRS. WILLIAM A. HAWLEY.

Mrs. Elizabeth S. Willard, wife of Dr. William A. Hawley, after an illness of several months, died June 23d. Mrs. Hawley was born in Lancaster, Mass., June 2d, 1820. She spent several years in Kentucky as a teacher, and was married to Dr. Hawley in 1851. In 1861 Dr. Hawley came to Syracuse, and during all the years since that time Mrs. Hawley has been known and loved by a wide circle of friends. Mrs. Hawley came of a well-known New England family of thoughtful and educated people, many of whom have been eminent as teachers and clergymen. She was a lady of fine endowments, good education, and extensive reading and observation, and has done, faithfully, her share in keeping up the standard of thinking and living among the people whom she knew. Three children survive, Mrs. Flora C. Howes, living in Holyoke, Mass., William A., of Pittsburg, Pa., and Miss Mary E. Hawley, of Syracuse.

